# Process evaluation of the ‘Grip on Health’ intervention in general and occupational health practice

**DOI:** 10.1186/s12913-022-08801-w

**Published:** 2022-11-30

**Authors:** Emma Vossen, Joost W. J. van der Gulden, Joost A.G.M. van Genabeek, Rosanne Schaap, Johannes R. Anema, Frederieke G. Schaafsma

**Affiliations:** 1 Department of Public and Occupational Health, Amsterdam UMC, Vrije Universiteit Amsterdam, Amsterdam Public Health research institute, Van der Boechorststraat 7, 1081 BT Amsterdam, Amsterdam, The Netherlands; 2 HAN University of Applied Sciences, Laan van Scheut 10, 6525 EM Nijmegen, The Netherlands; 3 Department of Primary and Community Care, Radboudumc, Geert Grooteplein Noord 21, 6525 EZ Nijmegen, The Netherlands; 4 TNO Work Health Technology, Schipholweg 77, 2316 ZL Leiden, The Netherlands

**Keywords:** Primary prevention, Quality of life, Occupational medicine, Primary health care, Interprofessional relations, Evaluation study, Socioeconomic factors

## Abstract

**Background:**

For working patients with a lower socioeconomic position, health complaints often result from a combination of problems on multiple life domains. To prevent long-term health complaints and absence from work, it is crucial for general and occupational health professionals to adopt a broad perspective on health and to collaborate when necessary. This study aimed to evaluate how the ‘Grip on Health’ intervention is implemented in general and occupational health practice to address multi-domain problems and to promote interprofessional collaboration.

**Method:**

A process evaluation was performed among 28 general and occupational health professionals, who were trained and implemented the Grip on Health intervention during a six-month period. The ‘Measurement Instrument for Determinants of Innovations’ was used to evaluate facilitators and barriers for implementing Grip on Health. Data included three group interviews with 17 professionals, a questionnaire and five individual interviews.

**Results:**

While most health professionals were enthusiastic about the Grip on Health intervention, its implementation was hindered by contextual factors. Barriers in the socio-political context consisted of legal rules and regulations around sickness and disability, professional protocols for interprofessional collaboration, and the Covid-19 pandemic. On the organizational level, lack of consultation time was the main barrier. Facilitators were found on the level of the intervention and the health professional. For instance, professionals described how the intervention supports addressing multi-domain problems and has created awareness of work in each other’s healthcare domain. They recognized the relevance of the intervention for a broad target group and experienced benefits of its use. The intervention period was, nevertheless, too short to determine the outcomes of Grip on Health.

**Conclusion:**

The Grip on Health intervention can be used to address problems on multiple life domains and to stimulate interprofessional collaboration. Visualizing multi-domain problems appeared especially helpful to guide patients with a lower socioeconomic position, and a joint training of general and occupational health professionals promoted their mutual awareness and familiarity. For a wider implementation, stakeholders on all levels, including the government and professional associations, should reflect on ways to address contextual barriers to promote a broad perspective on health as well as on collaborative work.

## Background

For employed patients with a lower socioeconomic position (SEP), health complaints often arise from an interplay of problems on multiple life domains [1–3]. For example, they are frequently faced with a combination of adverse working conditions, unhealthy lifestyles, financial problems and private or social issues [1–3]. According to a Dutch study, an estimated 13% of the population in the Netherlands suffers from multi-domain problems, versus 19% of people with a lower SEP [4]. Tackling problems on only one life domain is therefore often insufficient for the effective treatment of health complaints for this group of patients [2, 5, 6]. At the same time, lower SEP patients experience a lower ‘health literacy’; that is, they seem to have less “knowledge, motivation, and competences to access, understand, appraise, and apply health information in everyday life to make decisions regarding healthcare, disease prevention, and health promotion” [7]. These patients therefore face more difficulties in solving multi-domain problems on their own [2].

To prevent long-term health complaints and absence from work for lower SEP patients, it thus seems pivotal for both general health professionals (GHPs) and occupational health professionals (OHPs) to adopt a broad perspective on health and to address multiple life domains in consultations with their clients [2, 4, 8, 9]. Instead of viewing health as solely the absence of symptoms or illness, such a broader perspective fits with Huber et al.’s [10, 11] perception of ‘Positive Health’ as involving six dimensions: bodily functions, mental well-being, daily functioning, participation, quality of life and meaningfulness. Lower SEP patients seem to prefer a multidimensional perspective on health [6, 12, 13]. Globally, the World Health Organization (WHO) sees focusing on multi-domain problems as the task of both healthcare settings: primary health care “addresses the broader determinants of health and focuses on the comprehensive and interrelated aspects of physical, mental and social health and wellbeing” [8, 14] while occupational health “is an area of work in public health to promote and maintain highest degree of physical, mental and social well-being of workers in all occupations” [15]. Moreover, the WHO emphasizes the collaboration between general and occupational health practice in promoting the health of working patients [16].

However, although initiatives have been developed within general practice to address cross-domain issues, for example regarding poverty [8] or employment [17], whether or not this is actually the task of GHPs remains contested [8, 9, 18–20]. Moreover, strategies to systematically asses social determinants of health during consultations appear sparse [18, 21]. In the occupational health domain, a holistic approach of worker well-being is not yet an explicit part of its paradigm, although changes in the nature of work(places) and the workforce ask for such an approach [22–24]. Furthermore, little research efforts have been made to study the complex interplay between work- and non-work-related influences [24]. Finally, establishing interprofessional collaboration between general practitioners (GPs) and occupational physicians (OPs) appears problematic in practice, although ideally, both work together to avoid contradictory advices on work and health [2, 25–27]. In conclusion, a marked gap exists between the (global) recognition of the need to address multi-domain issues and the actual reality in general and occupational health practice.

Focusing specifically on occupational health, the ‘Grip on Health’ intervention was developed for OHPs as a way to address problems on multiple life domains, especially aimed towards lower SEP patients [2, 28]. Grip on Health adopts the six life domains of Positive Health and extends it with the structured, three-step Participatory Approach [29] in which the health professional and the person together identify and prioritize health problems, determine and select solutions, and evaluate outcomes. Please note that the Participatory Approach is a multidisciplinary guideline for conversations within occupational health practice, not to be confused with the participatory approach as a research methodology. In their evaluation study, Schaap et al. [30] showed that especially the visual materials of the Grip on Health intervention appeared useful to actively discuss and identify health problems and solutions. However, the specific occupational healthcare context complicated targeting lower SEP patients for early intervention as well as consistently applying the three steps of the intervention, due to, *inter alia*, a lack of priority to prevention and a lack of consultation time. The authors concluded that in order for interventions to successfully tackle multi-domain problems for patients with a lower SEP, involvement of GPs and better cooperation between OPs and GPs is necessary [2, 30].

The goal of the current study is therefore to evaluate how the Grip on Health intervention is implemented in both general and occupational health practice to address multi-domain problems and to promote interprofessional collaboration. More specifically, we evaluated the implementation process of the Grip on Health intervention in both healthcare domains, from the perspective of the health professional. We thereby focused on a broad range of GHPs and OHPs, as we assumed that these professionals might have more opportunities to apply the intervention compared to GPs and OPs. Moreover, we extended the Grip on Health intervention with the topic of interprofessional collaboration between GHPs and OHPs, further elaborating upon one of its key components to involve relevant stakeholders, such as an employer, partner or different health professional, after having identified the relevant life domain where problems occur [29].

## Methods

### Study design

To evaluate how the Grip on Health intervention was implemented in general and occupational health practice, a ‘process evaluation’ [31] was performed. Process evaluations using a systematic and mixed-method approach are important to understand how interventions work in practice [32]. Instead of only examining the intervention’s outcomes, process evaluations also consider context, process and mechanisms, answering the question “what works for whom under which circumstances?” [31–33]. This detailed examination provides insights with which the effectiveness of the intervention can be (further) improved.

We conducted the process evaluation by means of the validated ‘Measurement Instrument for Determinants of Innovations’ (MIDI) [34]. MIDI is particularly suitable for our study, as it has been developed specifically for the implementation of innovative health interventions and focuses on the perspective of health professionals. That is, MIDI is a generic diagnostic tool, developed by Fleuren et al. [34], to systematically map and investigate determinants of the intervention implementation process. The framework distinguishes 29 determinants related to the innovation, the user (i.e., the health professional), the organizational and the socio-political context. For each determinant, question(s) and response categories are indicated, as well as information on how to analyze the responses. The MIDI framework was developed on the basis of a literature review, a Delphi survey among implementation experts and a meta-analysis of empirical studies on the adoption of innovations in health care [34]. The framework is applicable to a broader range of settings. First, the original list of determinants stemmed from many different healthcare settings. Second, the experts involved in the development studies found that most determinants were generic. Third, since its publication in 2014, MIDI has been validated, used and tested in process evaluations of different types of healthcare interventions [35–38].

In the current study, we focused on MIDI’s determinants related to the innovation, the health professional and the socio-political environment. Since the Grip on Health intervention in our study was not implemented in specific organizations, we removed the determinants related to the organization (except for one) and to colleagues, as these determinants did not apply to the situation at hand. Moreover, one determinant regarding the professional obligation to ask about multiple life domains was removed since this topic was explicitly part of the training. Another determinant regarding awareness of the intervention’s content was removed since all health professionals participated in the training. This means that 15 of MIDI’s 29 determinants were selected. Table 1 provides an overview of these determinants and their definition, including the data collection method that was used. Data collection was performed between September 2021 and February 2022. Data stemmed from semi-structured (group) interviews and a questionnaire among health professionals participating in the Grip on Health intervention. This study is approved by the Medical Ethical Review Committee of the VU University Medical Center (number 2021.0332).

**Table 1:** MIDI determinants used in this study

Determinant	PCM 1/2	PCM 3	Questionnaire
*Intervention*			
1. Procedural clarity: Extent to which the innovation is described in clear steps/procedures			X
2. Correctness: Degree to which the innovation is based on factually correct knowledge			X
3. Completeness: Degree to which the activities described in the innovation are complete			X
4. Complexity: Degree to which implementation of the innovation is complex			X
5. Compatibility: Degree to which the innovation is compatible with the values and working method in place	X^a^	X^a^	X
6. Observability: Visibility of the outcomes for the user		X^b^	
7 . Relevance for client/patient: Degree to which the user believes the innovation is relevant for his/her client	X	X^b^	
*User / healthcare professional*			
8. Personal benefits/drawbacks: Degree to which using the innovation has (dis)advantages for the users themselves		X^a^	
9. Outcome expectations: Perceived probability and importance of achieving the patient objectives as intended by the innovation		X^b^	
11. Patient satisfaction: Degree to which the user expects patients to be satisfied with the innovation	X	X^b^	
12. Patient cooperation: Degree to which the user expects patients to cooperate with the innovation	X	X^b^	
16. Self-efficacy: Degree to which the user believes he or she is able to implement the activities involved in the innovation	X^a^		
17. Knowledge: Degree to which the user has the knowledge needed to use the innovation			X
*Organization*			
23. Time available: Amount of time available to use the innovation		X^b^	
*Socio-political context*			
29. Legislation and regulations: Degree to which the innovation fits in with existing legislation and regulations established by the competent authorities	X^c^	X^c^	

Source: [39]

*PCM *Peer consultation meeting. Determinant 10 (professional obligation) was excluded since this item was addressed during the training; determinant 18 (awareness of content of the innovation) was excluded because all respondents participated in the training; determinants 13–15 (regarding colleagues) and determinants 19–28 related to the organization (except item 23) were excluded. Determinants were literally copied from MIDI, except: ^a^response categories were altered to ‘thumbs up’ or ‘thumbs down’ in an interactive assignment; ^b^determinants were changed into open-ended questions; ^c^determinant was changed in different wording

### The Grip on Health intervention

The Grip on Health intervention was developed by Schaap et al. [28, 30]; in their publications a more detailed description of the development, content and first evaluation of the intervention can be found. In short, Grip on Health is a structured, three-step conversation method that helps to identify and solve multi-domain issues affecting work and life functioning. Central to this method is that it puts the person (i.e., the patient) in the lead. Another key element is the involvement of relevant stakeholders, such as life partners, employers or different health professionals like the GP or OP. This implies that instead of being the expert, the health professional takes on the role of process leader, who guarantees the equal and active input of all participants and aims to generate consensus about issues and solutions.

Answering the question ‘How are you doing?’, in the first step of Grip on Health, the health professional encourages the patient to identify life domains in which problems occur and to determine the most important problem(s) that need(s) to be tackled. Second, the health professional and the patient – and where necessary, relevant stakeholders – brainstorm about possible solutions for the problems selected in the first step, strive to reach consensus on these solutions and create a plan of action. The question ‘What can we do to improve your situation?’ is central to this step. Here, it may be necessary to involve a health professional working in the ‘other’ healthcare domain of occupational health or general practice and to collaborate on problems and solutions. This involvement is only possible after consent by the patient. In an ideal situation, these health professionals would join a meeting, but collaboration can also take place by telephone and, least preferable, by giving input on paper. Third, in the final step, the health professional and the person evaluate the plan of action and determine whether changes have to be made, answering the question ‘Has your situation improved?’. Materials are available to support each step, such as conversation forms and cards.

In September 2021, two groups of health professionals received a training in the Grip on Health conversation method. Each training consisted of a two-hour theoretical instruction and a three-hour practice meeting one week later. The instruction covered both the content and the theoretical background of Grip on Health (i.e., multi-domain problems, Positive Health, Participative Approach), as well as the *how*, *what* and *when* of interprofessional collaboration between occupational health and general practice. During the practice meeting, professionals practiced with the three steps of Grip on Health and with interprofessional collaboration. Authors FS and EV gave the theoretical instruction, while the practice meetings were held by EV and two teachers, who had a background as either OP or GP. We purposefully selected these teachers to explicitly bring together both professional disciplines. Because of the Covid-19 pandemic, the theoretical instruction was held online, using MS Teams. The practice meetings were held in two different locations. Afterwards, the professionals were instructed to start using Grip on Health during their consultations with patients with a lower SEP, which we defined as patients with a lower educational background, lower income and often performing manual labor [1].

Furthermore, in November and December 2021, three two-hour peer consultation meetings took place; professionals could choose which of the three consultation meetings they would attend. These meetings were held online by authors EV, RS and one or two of the aforementioned teachers. Finally, in February 2022, five professionals were invited for an evaluation interview. For most professionals, accreditation was granted, depending on their presence during the meetings. The training was given free of charge for professionals.

### Study population

Health professionals were recruited through repeated calls via the advisory committee of this research project, professional associations, networks of general practices, occupational health services and via LinkedIn. Professionals could sign up for participation with author EV. A purposive sampling strategy was used, since we aimed to include a specific set of health professionals: regarding OHPs, we recruited OPs, occupational health nurses, assistant practitioners (APs), practice nurses or case managers in occupational health, while regarding GHPs we enrolled GPs, general-care nurse practitioners (NPs), physician assistants (PAs), practice nurses in physical health care and APs in mental health care. No prior relationships existed between the authors and participating health professionals. Health professionals received an information letter approved by the Medical Ethical Review Committee and signed informed consent before participation.

Initially, 31 GHPs and OHPs took part in the training; however, three professionals stopped after the theoretical instruction because of either high workloads at their jobs (2) or the location of the practice meeting (1). This resulted in 28 professionals in the Grip on Health training, consisting of 18 OHPs and ten GHPs. Of the 28 professionals, 86% was women. Seventeen health professionals participated in one or two peer consultation meetings: respectively six, four and ten professionals were included in the three consultation meetings, and the latter meeting was split into two groups. The other 11 professionals did not participate in any of the consultation meetings due to either high workloads (4), the absence of accreditation (1), not having worked with the Grip on Health method (2), no-show after signing up (2) or not responding to invitation e-mails (2). Five professionals participated in an evaluation interview (see below). Table 2 provides an overview of the participating health professionals in the different meetings.

**Table 2:** Number and type of health professional in the Grip on Health intervention

Health professionals	Gender	Training	PCM 1	PCM 2	PCM 3^a^	Interview
*Occupational health professional*						
Occupational physician	3 F;1 M	4	1	-	3	-
Occupational health nurse	1 F	1	-	-	1	-
Asst. practitioner / practice nurse occupational health^b^	9 F;3 M	12	3	1	1	1
Case manager	1 F	1	1	-	-	1
*General health professional*						
General practitioner	2 F	2	-	-	-	-
General-care nurse practitioner	1 F	1	-	1	-	1
Physician assistant	1 F	1	-	1	1	-
Practice nurse physical health care	2 F	2	1	-	1	1
Asst. practitioner mental health care	4 F	4	-	1	3	1
*Total*	24 F;4 M	28	6	4	10	5

*PCM *Peer consultation meeting, *F *Female, *M *Male

^a^This peer consultation meeting was split into two groups (3a and 3b)

^b^The assistant practitioner / practice nurse occupational health is a relatively new occupation in the Netherlands (in Dutch: ‘praktijkondersteuner bedrijfsarts’). Previous education determines the content of the tasks of the assistant practitioner (non-medical background) or practice nurse (medically trained)

### Data collection

Data in our study consisted of both qualitative information (group and individual interviews) and quantitative information (questionnaire). First, besides a way to share experiences, the peer consultation meetings were used as group interviews. These interviews were structured by using determinants of MIDI; see Table 1 for an overview of the data collection method that was used for each determinant. Where MIDI is originally a quantitative method, during the consultation meetings we used it as an interview guide, to ensure that relevant domains of implementation were discussed, as is done in earlier studies [35, 38]. Questions were presented on-screen one by one and the professionals answered the questions in an interactive program (Teams Forms or Mentimeter), after which the answers were discussed and elaborated upon in the group.

Second, after the training took place, the 28 participating professionals were sent an evaluation questionnaire. Besides questions evaluating the content, organization and teachers of the training, the questionnaire consisted of the intervention- and user-related determinants of MIDI that were not asked during the group interviews (see Table 1). Twenty-two professionals filled out the questionnaire, resulting in a response rate of 79%, practically similar between the GHPs and OHPs.

Third, after the peer consultation meetings and the questionnaire, author EV held five evaluation interviews with professionals who participated in the training, in order to validate, clarify and add to our findings. To obtain different perspectives, we purposefully selected professionals who were either very positive about the method during the group consultation meetings (2), were more negative (1) or expressed little use of the method (2). Topics of the interviews entailed the professionals’ experiences with the application of Grip on Health in practice, the most important (dis)advantages of the method, an evaluation of when and why (not) to use its different components, and advice for improvement. Furthermore, we asked questions about the introduction and application of the method in the organization of the professional, to cover the organizational context. The interviews lasted approximately 30–45 min.

### Data analysis

Quantitative questionnaire data were analyzed by means of descriptive statistics in SPSS. The group interviews were audio-recorded (with permission) and transcribed verbatim, whereby the input of each professional was anonymized. Obtained data were stored in a secured digital environment and were only accessible to the authors. We used deductive thematic analysis [40] to analyze the transcripts, using MIDI as our framework. In our analyses, the focus was on the breadth of implementation determinants – barriers as well as facilitators – in general and occupational health practice. In a first step, the transcripts were read, summarized and coded by author EV, supported by the use of MAXQDA. This led to an initial list of codes. Another author (RS) read and coded one transcript, after which EV and RS discussed the code list to reach consensus on the codes. In a next step, EV collated codes into themes, guided by the determinants of MIDI (i.e., the determinants related to the health professional, the innovation, the organizational context and the socio-political environment). At this point, authors FS and JvG each read one transcript and reviewed the themes on accuracy. After that EV further refined the specifics of each theme. Finally, the individual interviews were summarized by EV in separate reports, according to the topic list. They were used as a way to reflect on and add to the findings of the group interviews.

## Results

Below, we describe the facilitators and barriers for implementation of the Grip on Health intervention on the level of the socio-political and organizational context, the intervention and the health professional. A summary of these facilitators and barriers is provided in Table 3. For readability reasons, we start with the contextual levels.

**Table 3:** Summary of facilitators and barriers for implementing Grip on Health (*n* = 28 health professionals, 2021, the Netherlands, results from group and individual interviews and questionnaire)

Determinants	Facilitator	Barrier
*Socio-political context*		• Covid-19 pandemic increases workload for GHPs, and related measures to work from home hinder using Grip on Health for OHPs
		• Legal rules and regulations for sickness absence hinder taking the role of process leader for OHPs
		• Professional protocols of task delegation for OHPs hinder interprofessional collaboration
*Organizational context*	• Enthusiasm within the organization for the adoption of Grip on Health	• Consultation times are too short for certain GHPs to apply Grip on Health as intended
		• Staff shortages decrease possibilities for implementation for GHPs
		• Agreements on interprofessional collaboration vary between practices
*Intervention-related determinants*	• Grip on Health is clear, based on factually correct knowledge, complete and easy to use	• Training is necessary for a correct use of Grip on Health
	• Grip on Health is especially relevant for professionals looking for a new methodology to address multi-domain problems	• Incompatibility with existing tools make Grip on Health an extra task
	• Grip on Health results in increased awareness among GHPs and OHPs	• Expected results of using Grip on Health are not yet clear and can only be determined in the longer term
	• Grip on Health is applicable to a large target group	• Lower SEP workers need guidance to use the method
*Determinants related to the health professional*	• Grip on Health has personal advantages: systematic and holistic approach that gives structure and guidance, insight and overview.	• Effects on patient satisfaction and cooperation are not yet clear • Grip on Health can be intimidating to patients when they are confronted with multi-domain problems.
	• Joint training of GHPs and OHPs increases mutual awareness and reciprocal familiarity necessary for interprofessional collaboration	• GHPs and OHPs do not have enough knowledge on *how* to collaborate
	• Addressing multi-domain problems is already part of the job for GHPs and OHPs	
	• GHPs and OHPs have the knowledge to apply Grip on Health as intended	

### Determinants related to the socio-political context (determinant 29)

#### The Covid-19 pandemic and related measures

**Fig. 1:**
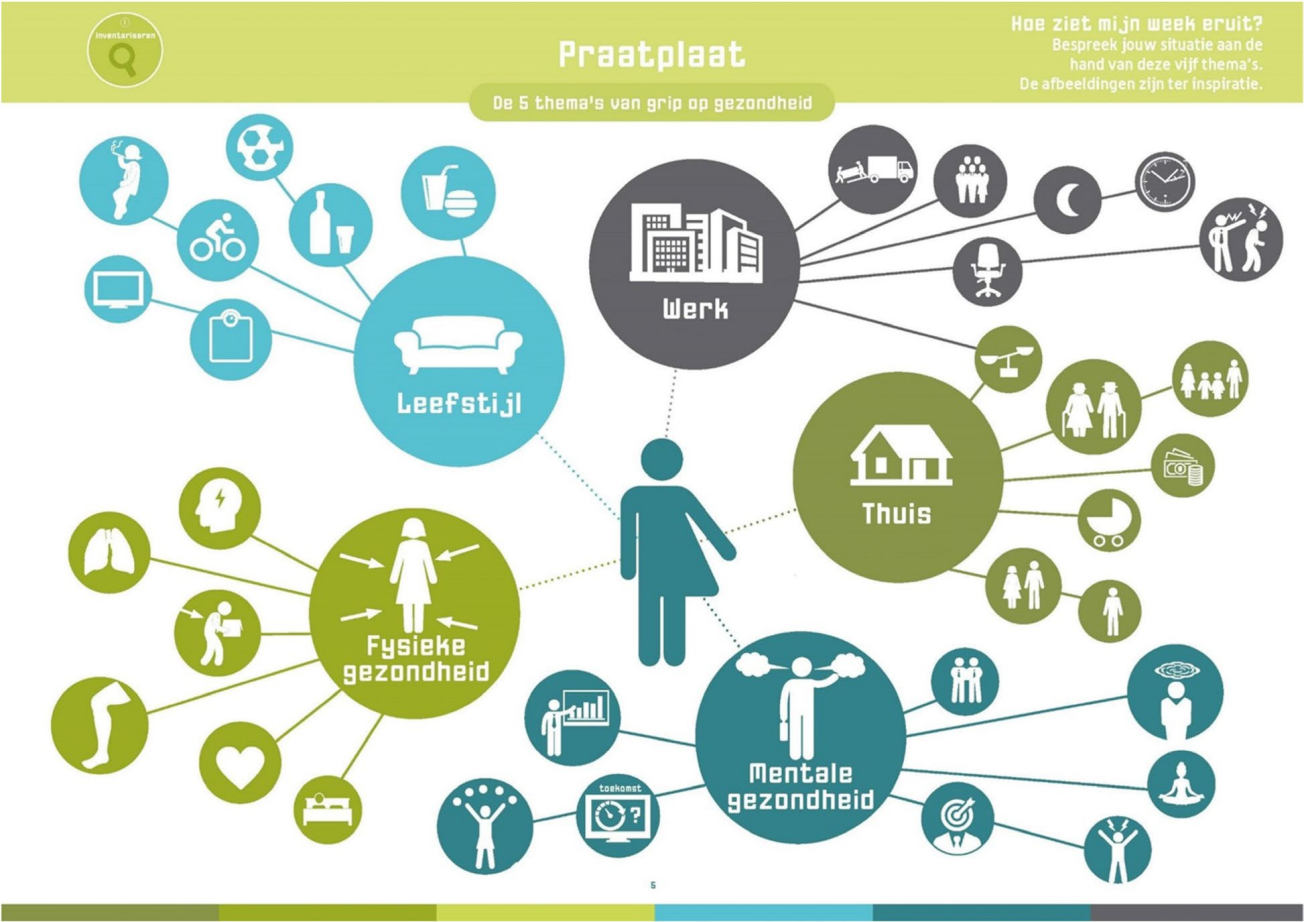
Visual conversation map

An important barrier for health professionals to implement the Grip on Health intervention appeared to be the Covid-19 pandemic and the related measures taken by the Dutch government. For GHPs, the pandemic resulted in higher workloads and therefore less time to apply the intervention. For OHPs, the regulation to work from home meant that consultations are conducted by telephone or online. Although a digital version of the materials was provided to the professionals, they thought it was too inconvenient or undesirable to share their screen to show the materials while being in an online meeting, or to show the materials in front of their screen while simultaneously having to point elements on the visual ‘conversation map’ (see Fig. 1). Professionals therefore agreed that Grip on Health is not suitable for online use, especially for the target group of lower SEP patients. However, although they could not use the materials of Grip on Health together with their patient, the intervention reminded them to address multiple life domains and some of them used the material (especially the conversation map) as a diagnostic tool. One AP in mental health said during a group consultation meeting:

I’ve worked from home, by telephone or online, and then I couldn’t use the materials. I do try to keep the concept in mind, asking about all life domains, choosing a focus and putting the patient in the lead (AP mental health, group interview 3b).

The Covid-19 pandemic is, nevertheless, not perceived as a lasting barrier to apply the intervention, when professionals work face-to-face again. During another group consultation meeting, an AP in occupational health described:

Now it’s more the situation that I can’t use it, but when I’m in the situation that I can talk to people face-to-face, then I will definitely use it (AP occupational health, group interview 1).

#### Legal regulations concerning sickness absence and disability

Although Grip on Health is intended to be applied in the preventive phase – before (long-term) sickness absence occurs – for OHPs, legal rules and regulations around sickness absence and disability influence how they implemented the intervention. This especially impacts the implementation of the focus on putting the patient in the lead and taking on the role of process leader instead of the expert. That is, according to the Dutch Gatekeeper Act, OPs in the Netherlands are legally obliged to give an advice on return to work and to record in a file that everything is done to this end. This is important to enable the employee, when necessary, to apply for a disability benefit after a sickness absence of two years and to prevent sanctions for employers. OHPs thus need to balance their expert, guiding role with the role of process leader that the Grip on Health intervention expects. For instance, when the employee chooses to first solve private issues, it is perceived as rather impossible to ignore the work domain. One OP and an AP in occupational health explained this as follows during a group consultation meeting:

What I notice when I identify problems on all life domains together with employees, is that they prioritize issues that are outside the areas of the Gatekeeper Act [i.e., non-work related]. And then I get in trouble with my [legal] deadlines, so some guidance is sometimes necessary (OP, group interview 1).

I recognize exactly what the OP [above] is saying. If someone mentions as a priority: “I can only return to work after my divorce problems have been solved”, and simultaneously there is an advice of the OP stating that someone can increase his or her working hours, then you have to say: “okay, we continue to build up your working hours” (AP occupational health, group interview 1).

#### Professional protocols of task delegation

A final barrier at the socio-political level for the implementation of Grip on Health is the professional protocol of task delegation by OPs, specifically regarding collaboration with GHPs. While Grip on Health emphasizes involving relevant stakeholders in discussing problems and solutions, the Protocol Task Delegation [41] prohibits OPs to delegate this collaboration to others. Case managers and APs in occupational health seemed to agree with this statement, saying that they are not medically trained and therefore do not want to get involved in medical discussions with GHPs. They therefore did not engage in collaborations with professionals in general care, as promoted by Grip on Health. One AP explained during a group consultation meeting:

I haven’t engaged in collaborations because I think that’s a task of the OP and not mine. So that’s the limitation of my job: this task has to be delegated to me before I can perform it on behalf of the OP. […] [But] I don’t think this is very common, because I’m not a medical professional. And you do often talk about medical information (AP occupational health, group interview 1).

### Determinants related to the organizational context

Although we did not focus on specific organizations, the group consultation meetings and the individual interviews revealed that the organizational context played an important role in how health professionals implemented Grip on Health. Some professionals told that their organizations were enthusiastic about the intervention and wanted to examine possibilities for its implementation; others mentioned how they taught Grip on Health to their colleague(s). On the other hand, for GHPs, applying the intervention was a challenge because of workloads that were already high due to a shortage of staff within their general practice and that increased due to the Covid-19 pandemic. Next, possibilities for interprofessional collaboration by GHPs with OHPs seemed to depend on agreements within the particular general practice. For example, during an interview, one general-care practice nurse described how she is allowed to contact OHPs, but that this was not the case in the practice where she worked before, since her former GP-employer wanted to be in control of collaborations with OHPs.

#### Time available: consultation time (determinant 23)

One organizational-level determinant that was mentioned very explicitly during the group consultation meetings was the time available to implement Grip on Health: consultation time appeared to be a barrier for its implementation. While OHPs, APs and practice nurses in general care experienced sufficient time to apply the intervention, GPs, PAs and NPs described that their consultation times of ten to 15 min are too short to complete the three steps. Professionals agreed that Grip on Health requires more than one consultation if all the steps are to be taken, but even the first step, where problems on different life domains are investigated, may ask for multiple consultations. For some professionals it is not standard practice that a patient has follow-up meetings. In that case, professionals themselves need to take action in scheduling a new consultation, which is sometimes perceived as impossible or as requiring too much effort (patients were not replying). Others described how they used step 1 as a preparation for the health professional that sees the patient next, for example a case manager for an OP or a NP for the AP for mental health.

Time wise I can’t finish it; I have 15 min per patient […]. I can also schedule a double consultation, but then I need to know this in advance, more time would be nice, but yes it’s quite busy in our practice so that’s difficult (PA, group interview 3b).

The advantage of occupational healthcare is that we have 45 min per consultation, so we can easily ask about all life domains (OP, group interview 3b).

### Determinants related to Grip on Health

#### Content: clarity, correctness, completeness and complexity (determinants 1–4)

Table 4 shows the results of the questionnaire regarding determinants 1–5 and 17. Overall, the majority of professionals evaluated the Grip on Health intervention as clear, based on factually correct knowledge, complete and easy to use, although OHPs are more positive than GHPs. However, during the group consultation meetings, the interviews and in the questionnaire, professionals mentioned how they would have liked more time to practice, both during and between the different meetings, so that they could obtain and share more experiences. Some professionals also mentioned that training in this intervention is necessary for it to be used correctly.

**Table 4:** Descriptive statistics for determinants used in the questionnaire (*n* = 22 health professionals, 2021, the Netherlands)

	**Total**						**Occupational health professionals**						**General health professionals**					
	*(Totally) disagree*		*Neither agree nor disagree *		*(Totally) agree*		*(Totally) disagree*		*Neither agree nor disagree*		*(Totally) agree*		*(Totally) disagree*		*Neither agree nor disagree*		*(Totally) agree*	
	n	%	n	%	n	%	n	%	n	%	n	%	n	%	n	%	n	%
The innovation clearly describes the activities I should perform and in which order (determinant 1).	2	9%	2	9%	18	82%	1	7%	-	-	13	93%	1	13%	2	25%	5	63%
The innovation is based on factually correct knowledge (determinant 2).	1	5%	6	27%	15	68%	-	-	3	21%	11	79%	1	13%	3	38%	4	50%
The innovation provides all the information and materials needed to work with it properly (determinant 3).	3	14%	2	9%	17	77%	1	7%	1	7%	12	86%	2	25%	1	13%	5	63%
The innovation is too complex for me to use (determinant 4).	16	73%	5	23%	1	5%	9	64%	4	29%	1	7%	7	88%	1	13%	-	-
The innovation is a good match for how I am used to working (determinant 5).	2	9%	4	18%	16	73%	1	7%	3	21%	10	71%	1	13%	1	13%	6	75%
I know enough to use the innovation (determinant 17).	1	5%	5	23%	16	73%	-	-	3	21%	11	79%	1	13%	2	25%	5	63%

Results of the questionnaire are only displayed for the MIDI determinants

#### A note on compatibility (determinant 5)

Grip on Health seems especially attractive for professionals looking for a (new) methodology to systematically address multiple life domains. That is, although almost 75% of the professionals (totally) agreed that Grip on Health is compatible with their current ways of working (see Table 4), lack of compatibility appeared an important barrier for implementation during the group consultation meetings and interviews. While the focus on identifying multi-domain problems matched the professionals’ ways of working, the forms of Grip on Health sometimes did not. Some OHPs mentioned that the forms to register problems on multiple life domains were not compatible with their existing checklists that identify the patient’s (in)abilities to work. These forms were seen as another ‘thing to do’ piling up on their existing workload. For some GHPs, in competition with current tools, Grip on Health needs to show distinctiveness and added value:

It needs to have added value, because we have a lot of tools that we use and then it needs to add something (nurse specialist, group interview 2).

#### Observability of expected results (determinant 6)

Given that the duration of the intervention was relatively short, the main result mentioned by the professionals during the group consultation meetings was their own increased awareness. This was revealed in three topics; namely, greater awareness of (1) a broad, multidimensional view on health, (2) the necessity for a different communication approach towards lower SEP patients, and (3) of work in the ‘other’ healthcare field of general or occupational health. Some professionals mentioned that they expected the intervention to be efficient because a detailed inventory of multi-domain problems in the beginning decreases the number of consultations. The professionals’ increased awareness is displayed in the following quotes:

It’s a different way of consulting from only focusing on numbers, blood sugar levels, weight, blood pressure, which are very important, but the context is equally important […] Eventually, it’s about the person behind the patient (practice nurse physical health, group interview 3b).

The realization that, hey, this woman belongs to the target group of lower SEP, that asks for a different approach than a higher educated nurse, for example. The [conversation map] has helped me in that sense (AP occupational health, group interview 2).

I’m more conscious in trying to collaborate with OPs, so you could see that as an effect and it also gives more structure to my consultations (AP mental health, group interview 3b).

#### Relevance for lower SEP patients (determinant 7)

Professionals agreed that Grip on Health is applicable for its target group of lower SEP patients. According to the professionals, especially the visual aspect of the intervention seems to fit with these patients, as they are often not that articulate. At the same time, according to the professionals, guidance is necessary for lower SEP patients, since some of the wording was perceived as too difficult (e.g., *mental* health and *physical* health) or they did not understand or resisted the assignments. As one NP mentioned during a group consultation meeting:

I can imagine that it is supportive or provides structure for people who are not that articulate, then I do see the added value (NP, group interview 2).

However, despite our instruction to apply the intervention to lower SEP patients, the professionals used Grip on Health for a wider group of patients. All professionals acknowledged that Grip on Health is applicable irrespective of a patient’s SEP, especially in case of psychological health complaints that ask for structure and overview, when patients are quieter or very talkative so that structure in the conversation is necessary.

### Determinants related to the health professional

#### Personal benefits/drawbacks (determinant 8)

Professionals see benefits of the Grip on Health intervention in their daily practice. Next to the abovementioned increased awareness, they mentioned how the intervention offers them a systematic approach that provides structure and guidance, and gives insight into and overview of (causes of) problems and solutions. Especially the visual aspect of the conversation map (see Fig. 1) contributes to these benefits. Another benefit is the holistic perspective of the intervention, which promotes a broad multidimensional view on health and a move away from sickness and curing to health and behavior. During a group consultation meeting, one PA described the benefits of structure and overview for herself as follows:

I found it very useful to prevent forgetting things and to make the picture as complete as possible (PA, group interview 2).

No personal drawbacks were mentioned.

#### Outcome expectations and professional obligation (determinant 9–10)

The goal of the Grip on Health intervention is to prevent long-term health complaints and sickness absence for lower SEP patients by (1) addressing problems on multi-life domains, and (2) increasing interprofessional collaboration between GHPs and OHPs. Due to the relatively short duration of the project and (measures related to) the Covid-19 pandemic, professionals did not (yet) experience these outcomes. During the training, professionals mentioned that addressing multi-domain problems is part of their job. The intervention supports their awareness of multi-domain problems, but in order to reach its ultimate goal, professionals feel that a paradigm shift is needed in both general and occupational health practice from focusing on sickness and curing to health and prevention. Professionals did, nevertheless, experience small changes in patients: Some professionals described how they see a change in the patient’s awareness, when addressing multiple life domains. By identifying and prioritizing issues and finding solutions, patients become activated and get a better grip on their situation.

For some patients, the penny dropped, as they said: “hey, these domains are all me. Hey, there’s a connection.” […] At once, it became clear that everything affects and is affected by everything (practice nurse physical health, group interview 1).

I keep it small, but the fact that just getting a patient to think and take on an active role, even that’s an effect (practice nurse physical health, group interview 3b).

It just works: […] making the patient mainly responsible, that they can take small steps, which stimulates and motivates them to make a next step, to celebrate their successes and become healthier that way (AP mental health, group interview 3b).

Regarding interprofessional collaboration, many professionals said that the intervention did not (yet) change their ways of working, while few increased the extent of collaboration. For instance, one physical health nurse described during an interview how she normally collaborates with OHPs once a year, while now she had about nine contacts during the training period. Still others, only made small changes by either providing patients with their business cards or write their telephone number on letters to an OHP or GHP with the explicit invitation to contact them. Nevertheless, all professionals agreed that a joint training for GHPs and OHPs helped to increase their mutual familiarity and helped to address reciprocal prejudices.

#### Satisfaction and cooperation of lower SEP patients (determinants 11–12)

Given the limited experience with the application of the intervention, for professionals it was difficult to assess the level of satisfaction and cooperation of lower SEP patients. According to the professionals, some patients apparently reacted positively to the intervention and appreciated being seen as a whole person. One physical health nurse described how a patient was relieved to be able to talk about multiple life domains, saying “I did not know I was allowed to talk about this too”. Other patients were described by the professionals as being more hesitant, especially when OPs asked about life domains other than work, as they were worried about the purpose of asking this information. Some professionals mentioned that patients did not like being given homework or thought the visual materials were childish. Other professionals talked about the necessity to put the visual map on the table beforehand, to prevent patients from thinking that something is so wrong with them that a special treatment is needed. Finally, professionals agreed that the intervention can seem intimidating to patients when they are confronted with problems on several levels, as an AP in occupational health described during a group consultation meeting:

[The intervention] is quite extensive and I think it can be quite intimidating. […] Because when you have problems on multiple domains, I can imagine it’s quite confrontational that you have to say that it’s not going so well on all domains (AP occupational health, group interview 3a).

#### Self-efficacy and knowledge (determinant 16–17)

Almost 75% of the professionals noted in the questionnaire that they have enough knowledge to apply the intervention as intended (see Table 4). However, the group consultation meetings showed that overall, Grip on Health is mainly referred to as a way to identify problems on multiple life domains by means of the visual map (i.e., step 1). GHPs mentioned that this step made it easier to ask about the domain of work, while OHPs told us that the intervention may provide a visual support (for themselves and patients) to address multi-domain problems, which they were already used to do. Step 2 and 3 were not frequently performed during the duration of the project. This was partly because of a lack of time, partly because it was not seen as necessary in the particular cases, and partly because of lack of experience with applying the intervention. While the visual map was received positively, professionals held mixed opinions about the conversation cards. Some professionals did not see a way to integrate the cards in their consultations, whereas others used them as a way to put the person in the lead. Finally, regarding interprofessional collaboration, the questionnaire shows that professionals know *when* to collaborate but still have questions regarding *how* to do so. They would have liked to receive more training on this topic, for instance “more practical ways to improve the contact between occupational and general health practice”, as one professional suggested in the questionnaire.

## Discussion

This study aimed to systematically asses implementation determinants (facilitators and barriers) of the Grip on Health intervention from the perspective of both GHPs and OHPs, with a special focus on interprofessional collaboration between both healthcare domains. The findings reveal that Grip on Health can be useful for health professionals to address problems on multiple life domains for lower SEP patients (and beyond) and increasing interprofessional awareness and familiarity. Particularly the structured and visual aspects of the intervention were valued by the professionals, as well as the joint training of GHPs and OHPs. First indications show that the Grip on Health intervention results in increased awareness among GHPs and OHPs of (1) the necessity to address multi-domain problems, (2) of work in each other’s healthcare domain and (3) of the importance to tailor communication styles to lower SEP patients.

### Facilitators and barriers for implementing Grip on Health

Although the implementation of the Grip on Health intervention was mainly facilitated by factors related to the intervention and the health professional, barriers were mostly found in the organizational context and on the socio-political level, in (temporary) governmental measures related to the Covid-19 pandemic, legal rules and regulations and professional protocols. Research suggests that the context, or “anything external to the intervention that may act as a barrier or facilitator to its implementation” [42], may be more important for the implementation of an intervention than its design and content [2, 43]. In this study, the importance of context showed both in addressing multi-domain problems and in interprofessional collaboration. We describe these issues and their implications below.

#### Addressing issues on multiple life domains

##### Barriers for GHPs

For most GHPs, the possibility to apply Grip on Health and address multi-domain problems was particularly influenced by the organizational context: a shortage of personnel and lack of consultation time decreased their opportunities to talk about domains other than health, and especially about work-related issues. These barriers are also recognized in earlier research regarding the attention for work in general practice [25, 44, 45]. Nevertheless, while our study underlines this finding for most GHPs, this was not the case for practice nurses in physical health and APs in mental health. In the Netherlands, these nurses and APs are working under the responsibility of a GP and generally have more consultation time than GPs. Given the increasing attention for the role of Dutch GPs in work-related health care (in the Netherlands, sickness certification is a task of the OP) [25, 44], our findings confirm earlier suggestions to delegate this task to practice nurses in physical health and APs in mental health [44, 46]. During their consultations they could, for example, make use of an evidence-based protocol with five questions for the early detection of work-related health complaints that may cause long-term absence from work [47].

##### Barriers for OHPs

By contrast, for OHPs, addressing multi-domain problems was already part of their jobs. However, since occupational health services in the Netherlands are reimbursed by employers who often prioritize sickness absence management over prevention, OHPs mostly come into the picture when employees are already (long-term) absent from work [2, 30, 48, 49]. At that point, our study revealed that legal rules and regulations for sickness and disability at the socio-political level determine actions and deadlines for OHPs. These legal frameworks appeared to pressurize the possibilities for OHPs to put the patient in the lead by taking on the role of process leader instead of the expert, and to arrive at shared decisions. This implies that for a correct implementation of the Grip on Health intervention – which focuses on the prevention of health complaints – employees should be reached earlier, when health issues arise but employees are at work and there are still options for OHPs to take on the role of process leader. The previous requires employers to focus more on prevention and to involve OHPs at an earlier stage.

#### Promoting interprofessional collaboration

##### Barriers for collaboration

Contextual barriers for interprofessional collaboration were in our study mainly present for OHPs. For them, interprofessional collaboration with GHPs was obstructed by professional protocols around task delegation at the socio-political level. These protocols dictate that interprofessional collaboration remains a task of the OP that cannot be delegated [41]. This implies that OHPs, other than OPs, are thus not allowed to collaborate with GHPs at the level of an individual patient.

On the other hand, some OHPs did not seem to feel responsible to have conversations with GHPs. That is, in the Netherlands, case managers and APs in occupational health are not required to have a medical background; the presence or absence of this background determines the tasks they are allowed to perform [41]. In the absence of a medical education, their task is limited to guiding employees with health problems to stay at, or return to work according to their remaining work capacity (determined by the OP), and they are not allowed to perform medical tasks. As a result, the case managers and APs in occupational health in our study do not identify themselves as healthcare providers for whom detailed medical information and treatment are relevant in relation to the management of the return-to-work process (and hence, they collaborate with workplace stakeholders rather than with other health professionals).

The above barriers imply that, in order to promote interprofessional collaboration between GHPs and OHPs, professional associations of OPs and other OHPs need to reconsider the task of interprofessional collaboration, taking into account what information is relevant for each health professional. That is to say, interprofessional collaboration does not have to be based on the exchange of medical information, but may instead be about the patient’s needs for a sustainable (work) functioning. Another possibility is to rethink which professional is in the best place to optimally participate in interprofessional collaboration (see also [46]).

##### Joint training as facilitator

Furthermore, while earlier research revealed a lack of knowledge about each other’s work and prejudices as important barriers for interprofessional collaboration between GHPs and OHPs [26, 27, 46, 50], we found the Grip on Health intervention to be a facilitator in this respect. Our study shows that particularly the training in Grip on Health has resulted in increased awareness and mutual familiarity and helped to address reciprocal prejudices. The participating health professionals were most enthusiastic about the coming together of different disciplines and perspectives, the possibility to get to know and learn from each other. Although we could not (yet) establish actual effects on the extent of collaboration, our findings suggest that interprofessional collaboration may be promoted by the joint training of GHPs and OHPs. Moreover, jointly training GHPs and OHPs already during their educations (i.e., ‘interprofessional education’, or IPE) may be the way forward to stimulate working interprofessionally [26, 51], provided that it is integrated in daily work practice [52].

### Contextual barriers in light of the Dutch healthcare system

The contextual barriers found in this study illustrate the general absence of incentives to cross healthcare domains and to collaborate interprofessionally within the Dutch healthcare system, resulting from its unique division between (the financing of) medical care and occupational medicine [48]. On the one hand, this division causes a lack of impetus (such as spending consultation time) to pay attention to ‘work’ within general practice [48, 49]. This is not only troublesome since people often contact their GPs first when health problems arise [2, 8, 17, 30], but also for the growing group of self-employed and temporary workers who have no access to occupational health care [48, 49]. On the other hand, since Dutch employers are responsible for occupational health, the care that OHPs can deliver depends on services purchased; contracts often appear to leave little room for prevention and tackling multi-domain problems [2, 48, 49]. These barriers call for the government to rethink the design and financing of the healthcare system, in order to promote interprofessional collaboration and a broad perspective on health. Doing so would also meet the global strategic principles of the WHO to “deliver occupational health in the context of integrated primary health care” [16].

### Strengths, limitations and recommendations for future research

A strength of our study is the systematic evaluation of the implementation of the Grip on Health intervention by health professionals, using the validated methodology of MIDI. Applying a process evaluation based on MIDI enabled to not only assess the results of the intervention, but also the determinants of the socio-political and organizational context, the intervention and the health professional that facilitate or hinder its implementation. Furthermore, the combination of qualitative (individual and group interviews) and quantitative evidence (questionnaire) enabled the triangulation of data, deepening our understanding and strengthening our conclusions [53].

Despite these strengths, MIDI takes the perspective of the user of the innovation [34, 39], which limits the evaluation of the implementation process of Grip on Health in our study to the perspective of GHPs and OHPs. We have attempted to address this issue by including lower SEP patients via the participating GHPs and OHPs, but unfortunately failed to do so. Some of the professionals mentioned that patients were hesitant to cooperate. The difficulty of engaging especially lower SEP patients in research has been mentioned before, in relation to a lack of trust between researcher and participant [54]. For future research, a recommendation is to build trustful relationships with ‘gatekeepers’ and patients by being physically present in the occupational health service or general practice. As Emmel et al. [54] called it: “being there and being seen”.

Relatedly, during the training and group consultation meetings we received comments that the Grip on Health materials, although specifically designed for lower SEP patients, still contain some wording that is too difficult for these patients. While recognizing that “lower SEP” is not a single population [55], it would be recommendable to further revise and test the materials together with the target group, in cooperation with a designer. Possibilities for the online use of the materials could then be investigated as well.

A second limitation of our study is the relatively short duration of the intervention. At multiple times we noticed that professionals had too little time to apply the intervention, which was further problematized by the Covid-19 pandemic. Professionals therefore experienced difficulties answering some of our questions, especially those relating to the outcomes and effects of Grip on Health. Moreover, the duration of the intervention appeared too short for Grip on Health to prove its added value in the ‘competition’ with other tools. This may have influenced our findings, for example regarding the (in)compatibility with existing tools. Future research could investigate the actual effectiveness of Grip on Health for the prevention of long-term health complaints and absence from work, as well as for the extent of interprofessional collaboration between GHPs and OHPs. This would make a(n even) greater case for the implementation of Grip on Health as a way to address multiple-life domains and collaborate in the prevention of health complaints and the sustainable employability of lower SEP patients.

## Conclusion

This study evaluated the process of implementing the Grip on Health intervention in general and occupational health practice, from the perspective of the health professionals. While factors related to the intervention and to the health professionals mainly facilitated implementing Grip on Health, its implementation was simultaneously hindered by contextual factors, such as governmental measures, legal and reimbursement frameworks, professional protocols and subsequent current ways of working (i.e., consultation time, existing tools and personnel shortages). This evaluation study reveals how a structured, stepwise and visual conversation method, Grip on Health, can support professionals in general and occupational health practice to address problems on multiple life domains of lower SEP patients and, if necessary, to collaborate with professionals in each other’s domain. The final outcomes of Grip on Health with regard to the prevention of long-term health complaints and absence from work of lower SEP patients as well as the extent of interprofessional collaboration nevertheless remain to be assessed in the future. In addition, for a wider implementation, stakeholders on all levels, including the government and professional associations, should reflect on ways to address contextual barriers to promote a broad perspective on health as well as collaborative work.

## Data Availability

The data in the current study are available from the corresponding author on reasonable request.

## Abbreviations

AP: Assistant practitioner
GHP: General health professional
GP: General practitioner
IPE: Interprofessional education
MIDI: Measurement instrument for determining innovations
NP: Nurse practitioner
OHP: Occupational health professional
OP: Occupational physician
PA: Physician assistant
SEP: Socioeconomic position
WHO: World Health Organization
